# New insight into relaxation dynamics of an epoxy/hydroxy functionalized polybutadiene from dielectric and mechanical spectroscopy studies

**DOI:** 10.1007/s00396-014-3254-4

**Published:** 2014-05-23

**Authors:** S. Hensel-Bielowka, Z. Wojnarowska, J. Knapik, M. Paluch

**Affiliations:** 1 Institute of Chemistry, University of Silesia, Szkolna 9, 40-006 Katowice, Poland; 2 Institute of Physics, University of Silesia, Uniwersytecka 4, 40-007 Katowice, Poland

**Keywords:** Dielectric spectroscopy, Liquid-glass transition, Relaxation dynamics

## Abstract

Dielectric and mechanical spectroscopy methods have been employed to describe the temperature dependencies of the segmental and macromolecular relaxation rates in epoxy/hydroxy functionalized polybutadiene. Dielectric studies on the dynamics of segments of the polymer as well as the mobility of small ions trapped in the system have been carried out both as a function of temperature and pressure under isobaric and isothermal conditions, respectively.

## Introduction

The non-Arrhenius temperature dependence of the molecular or segmental (in the case of polymers) relaxation times and the non-Debye character of related relaxation functions are still considered to be not fully understood features of dynamics, both in supercooled liquids and in polymer melts. A large number of spectroscopic and relaxation methods have been used to detect and characterize these phenomena in a very large number of versatile systems [1, 2]. In particular, information sourced from a variety of experimental methods seems to be helpful in categorizing and understanding these relaxation mechanisms. Among other methods, dielectric spectroscopy is systematically used to study the dynamics of polymer melts because of a broad range of accessible frequencies and a high sensitivity, both of which enable very accurate measurements [3, 4]. Multiple relaxation peaks can usually be distinguished in the dielectric spectra of dipolar polymers [5–7]. Their positions depend on temperature; pressure; and in some cases, the length of their chains. Many polymers have been extensively studied using this method. In this paper, we employ both dielectric and mechanical spectroscopies in order to study polymer melt dynamics, which consist of various relaxation modes distributed across a broad time scale.

The experimental methods used for the analysis of these relaxation phenomena examine different aspects of molecular motions. Dielectric response results mainly from the reorientation of permanent dipole moments, whereas translational segmental mobility gives the main contribution to mechanical spectra.

A lot of experimental work has been devoted to the study of temperature effects on the dynamics of polymer melts, whereas dynamic properties can be also controlled by a change of pressure. Nowadays, pressure is quite frequently used for this purpose despite the experimental difficulties which are involved [8–11]. The importance of pressure effects lies in the fact that pressure is the dominant thermodynamic variable responsible for changes in intermolecular distances, while temperature controls both the energy and density of a system. Pressure thus provides complementary information on the dynamics that allow the separation of kinetic effects from the effects that are associated with intermolecular interactions. In this work, we have compared the influence of temperature and pressure on the relaxation processes of epoxy/hydroxy functionalized polybutadiene (EHPB) determined from dielectric and mechanical measurements.

## Experimental

### Material

**Scheme 1:**
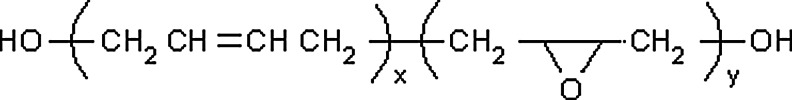
The chemical structure of EHPB

Epoxy/hydroxy-functionalized polybutadiene (EHPB) with a molecular structure as follows: 55 %—1,4–trans; 15 %—1,4–cis; and 30 %—1,2 vinyl and with a weight-average molecular weight *M*_w_ = 2,600 and polydispersity of two was purchased from Aldrich Chemicals. The number of epoxide units is defined by E.W. = 460. The chemical structure of EHPB is shown in Scheme 1. The glass transition temperature, *T*_g_ = 218 K, was determined from the middle point of the differential scanning calorimetry (DSC) curve recorded at cooling with 10 K/min.

### Methods

#### Mechanical spectroscopy

Dynamic mechanical measurements were taken by means of the Rheometrics RMS 800 mechanical spectrometer. Shear deformation was applied under conditions of controlled deformation amplitude, always remaining in the range of the linear viscoelastic response of the studied samples. Frequency dependencies of the storage (G′) and loss (G″) shear modulus were determined at various temperatures. Parallel plate geometry was used below 25 °C, with plate diameters of 6 mm, whereas at higher temperatures, the measurements were taken using cone-plate geometry, with diameters of 25 mm. In the case of plate-plate geometry, the gap between the plates (sample thickness) was about 1 mm. The experiments were conducted in a dry nitrogen atmosphere.

**Fig. 1:**
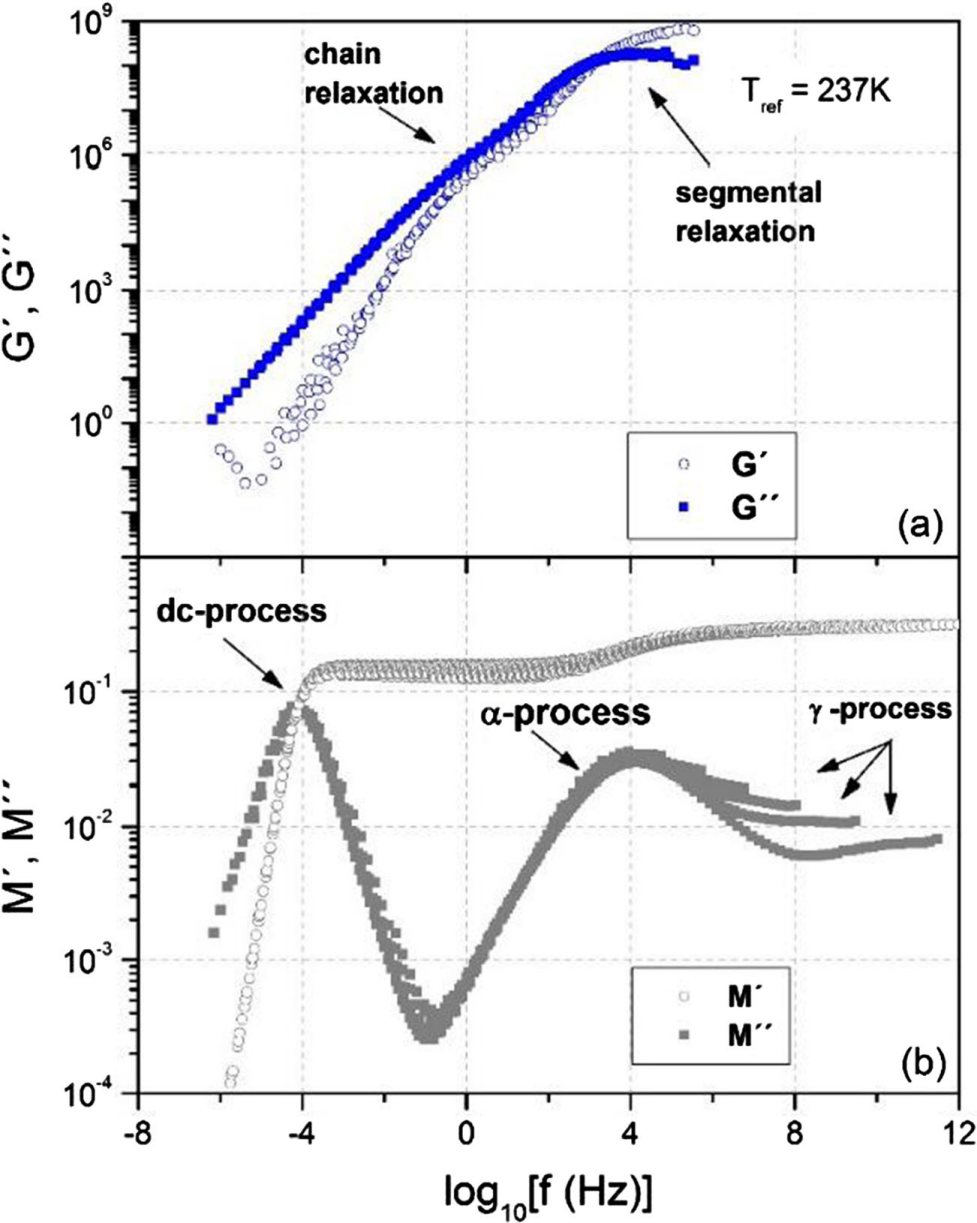
The master plot of the frequency dependencies of **a** the real (G′) and imaginary (G″) parts of the shear modulus and **b** the real (M′) and imaginary (M″) parts of the dielectric modulus determined by means of mechanical and dielectric measurements, respectively. The *master curves* in both cases correspond to the same reference temperature (*T* = 273 K) and are constructed with horizontal shifts only

Frequency dependencies of G′ and G″ measured within the range 0.1–100 rad/s at various temperatures were used to construct master curves. Only horizontal shifts were performed. This procedure provided a temperature dependence of shift factors (log *a*_*T*_ vs. *T*). The low-frequency range of the master dependence of G″ (with G″ ~ ϖ, indicating the Newtonian flow range) was used to determine the zero shear viscosity at the reference temperature (*η*_*o*_(*T*_*ref*_) = *G* ′ ′/*ϖ*). Viscosity values related to other temperatures have been determined as *η*_*o*_(*T*) = *η*_*o*_(*T*_*ref*_) + log *a*_*T*_. The relaxation time corresponding to the transition between the Newtonian flow range at low frequencies and the range of intramolecular relaxation at higher frequencies signified by a decreased slope of G′ and G″ was determined as *τ*_*c*_(*T*_*ref*_) = 1/*ω*_*c*_, where *ω*_c_ is the frequency at which the G′ and G″ dependencies extrapolated from the flow range cross each other (see Fig. 1a). The transition to a glassy state at the highest frequencies at the reference temperature was determined as *τ*_*s*_(*T*_*ref*_) = 1/*ω*_*s*_, where *ω*_s_ is the frequency at which the G′ and G″ dependencies cross each other. Relaxation times at other temperatures are given by *τ*(*T*) = *τ*(*T*_*ref*_) + log *a*_*T*_.

#### Dielectric spectroscopy

Dielectric spectroscopy measurements were carried out using an experimental set-up made by Novocontrol. The system was equipped with a Solartron impedance/gain phase analyzer SI 1260 and a broadband dielectric converter. Measurements were taken in the frequency range 10^−2^–10^6^ Hz at various temperatures. The sample, in a viscous liquid state at room temperature, was placed in a parallel plate cell with a diameter of 20 mm and a thickness of 0.1 mm. The temperature was controlled between 150 and 300 K using a nitrogen-gas cryostat. The temperature stability of the sample was better than 0.1 K.

For high-pressure measurements, a system constructed by UNIPRESS with a special flat-parallel capacitor described in detail in ref. [12] was used. The pressure-transmitting liquid was a mixture of heptane and silicone oil, and the pressure was measured by a Nova Swiss pressure tensometer with a resolution of 0.1 MPa. The sample was totally isolated from the medium delivering the pressure and was only in contact with stainless steel, Teflon, and quartz. The temperature was maintained within 0.1 K by means of a liquid flow provided by a thermostatic bath.

The results of the dielectric measurements are shown in two representations: (1) the traditional one, as a complex dielectric permittivity, ε*(ω) = ε′(ω) − iε″(ω) and (2) as a complex dielectric modulus M*(ω) = M′(ω) + iM″(ω). The latter, when considered in a form suitable for comparison with the results of the dynamic mechanical measurements, should be related to the dielectric permittivity as M*(ω) = 1 / ε*(ω). Thus, the modulus relates the external electric field (E) and the polarization (P) in the sample as E⋅ε_o_ = M* ∙ P in a similar way to the relation describing the viscoelastic properties (where the external shear stress (*σ*) is related to the shear strain (*γ*) by means of the complex shear modulus G* (*σ* = G* ∙ *γ*)). Relaxation times in each case have been determined as *τ* = 1/*ω*, where *ω* denotes the frequency of the maximum of the α-peak [13–16].

Although in principle both representations are equivalent, it still remains a contentious issue which one provides the best insight into dynamics. In a number of papers, Richert and Wagner placed particular significance on the fact that susceptibility refers to a retardation process, whereas modulus is related to a relaxation process [17]. This implies that there is a difference between the values of retardation and relaxation times, which will be evidenced in the subsequent part of this work. Actually, by simple Debye process, it can be easily shown that the ratio τ_ε_/τ_M_ is equal to ε_s_/ε_∞_, where ε_s_ and ε_∞_ denote the unrelaxed and relaxed parts of ε′, respectively. Analogous relationship is obeyed for mechanical data. For the non-Debye process, the difference between both time constants can be much greater.

## Results and discussion

As was signaled in the experimental section before comparing the results obtained by both the mechanical and dielectric methods, uniform representation have to be chosen. In the linear response regime, the dielectric and mechanical response are completely characterized by dynamic susceptibilities, i.e., mechanical shear compliance—J and dielectric permittivity—ε. Dielectric and mechanical response functions can also be expressed in terms of another generalized quantity called a rigidity (mechanical modulus—G* = 1 / J* and electric modulus—M* = 1 / ε*).

Figure 1a, b shows a comparison of master plots obtained by a superposition of frequency sweeps of the mechanical and dielectric moduli, respectively, recorded at various temperatures to a spectrum obtained at a reference temperature which is the same for both methods. It can be seen that a number of relaxation processes occur in the studied material and that they are distributed over a broad frequency range. In the mechanical spectrum, there are two processes. They can be attributed to segmental and chain relaxation, at high and low frequencies, respectively (Fig. 1a). The relaxation of polymer chains is detectable only in the mechanical method, where it controls the viscous flow of the material at low deformation rates. On the other hand, the segmental relaxation observed mechanically has its clearly seen equivalent in the dielectric spectrum, denoted as the α-process (Fig. 1b). In the dielectric spectra of EHPB, a few more relaxation processes can be seen. The slowest one, observed as a terminal single Debye process positioned at *ω* = *σ* / ε_s_ε_0_, is attributed to the ionic mobility in the system (dc-conductivity) (Fig. 1b). On the other hand, at frequencies higher than the α-process, faster relaxation peaks can be seen. They are called β and γ relaxations or more generally secondary processes.

**Fig. 2:**
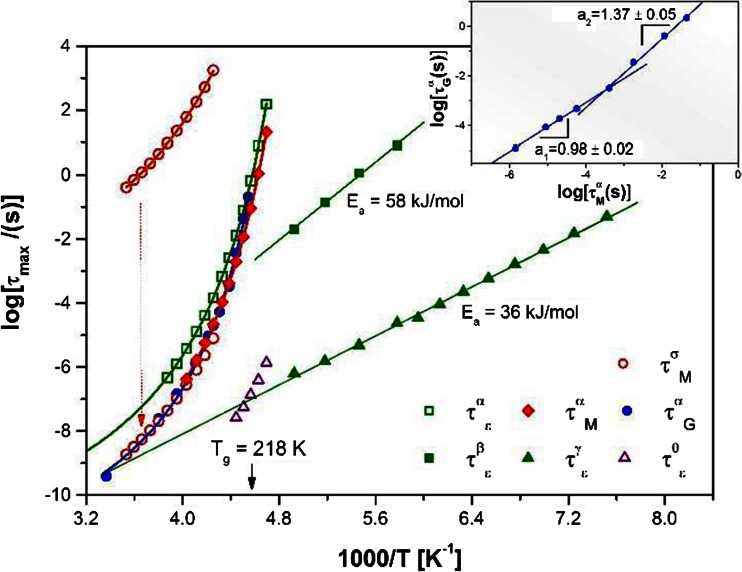
Relaxation times of various relaxation processes as a function of reciprocal temperature detected using the two experimental techniques. The *insert* shows a direct comparison of the segmental relaxation times as determined by means of dielectric and mechanical methods. The *arrow* indicates the way the ionic conductivity times are shifted to collapse onto the α-relaxation data

In order to explore possible couplings between various relaxation processes, the relaxation times of all modes have been collected in Fig. 2. As a first step, we would like to focus on the analysis of the temperature dependencies of the segmental relaxation rates determined from both dielectric and mechanical measurements. The temperature dependence of the relaxation times for the α-process is frequently parameterized by means of the Vogel-Fulcher-Tammann relation [18–20]:

1 $$ \tau ={\tau}_0 \exp \left(\frac{D_T{T}_0}{T-{T}_0}\right) $$

In Fig. 2, it is clear that in the case of the data obtained in both the dielectric and mechanical experiments, Eq. 1 can be successfully used. However, what is more interesting, at higher temperatures, both methods provide the same temperature characteristics for segmental mobility. On the other hand, the agreement between them is rather poor at lower temperatures. Approaching the glass transition, mechanical relaxation times start to systematically deviate from dielectric ones. Such behavior can result from the fact that the translational mobility of segments within polymer chain is much more of an impediment than the rotation of the permanent dipole moment near the glass transition temperature. A straightforward comparison of both relaxation times is shown in the inset to Fig. 2. Indeed, τ^α^_G_ increases faster than τ^α^_M_ in the vicinity of *T*_g_.

To illustrate how important the choice of uniform representation is when comparing the mechanical and dielectric data, in Fig. 2 we also plotted the temperature dependence of retardation times for the α-relaxation process. It is clear that relaxation times are shorter than the corresponding retardation times. In this case, the ratio τ^α^_ε_ / τ^α^_M_ = 5 is greater than ε_s_ / ε_∞_ = 2.

The low frequency effects in both the mechanical and dielectric spectra, although very similar, do not formally coincide in frequency and therefore must be related to completely different relaxation phenomena. In the mechanical spectra, the terminal Newtonian flow, characterized by G′ ~ ω^2^ and G″ ~ ω, is determined by the relaxation of polymer chains and is stretched out into the low-frequency regime below the frequency corresponding to the reciprocal value of the longest relaxation time. This relaxation time determining the range of the Newtonian behavior of the studied system is obtained from the cross-point between log_10_G′ and log_10_G″ vs. log_10_ω lines extrapolated to the higher-frequency range.

In the dielectric spectra, the regime of M′ ~ ω^2^ and M″ ~ ω is also observed but must be attributed to a current of ionic charge carriers. They are trapped in some way at frequencies corresponding to the plateau of M′ (higher frequencies) and become mobile at frequencies below the cross-point of M′ and M″. The reciprocal value of the frequency at which the M′ and M″ cross each other is taken as the relaxation time corresponding to a mobilization of ions. For a Debye process, this frequency corresponds to the M″ peak maximum and to the cross point between lines of log_10_M′ and log_10_M″ vs. log_10_ω when extrapolated to higher frequencies.

On the basis of classical theories of viscous flow and dielectrics, a correlation should exist between the segmental relaxation time of polymers and the electrical conductivity relaxation time of ions present in a system [21]. Therefore, the time constant of conductivity relaxation (τ^σ^_M_) can provide new information concerning the segmental relaxation/dynamics of the studied polymer. The temperature dependence of τ^σ^_M_ in EHPB is shown in Fig. 2 (with open circles). It is clear that the experimental data deviates from the Arrhenius-type dependence. In this case, a Vogel-Fulcher-Tammann (VFT) equation (Eq. 1) also gives a satisfactory description of τ^σ^_M_(T) dependencies with the following sets of parameters: logτ_0_/s = −4.89 ± 0.07, *D*_T_ = 6.4 ± 0.35, and *T*_0_ = 175 ± 2 K. A good coincidence of τ^σ^_M_(T) and τ^α^_M_(T) dependencies was again only found at higher temperatures. And only in this temperature region can τ^σ^_M_(T) data be used to extend the range of frequency and temperature for segmental relaxation.

**Fig. 3:**
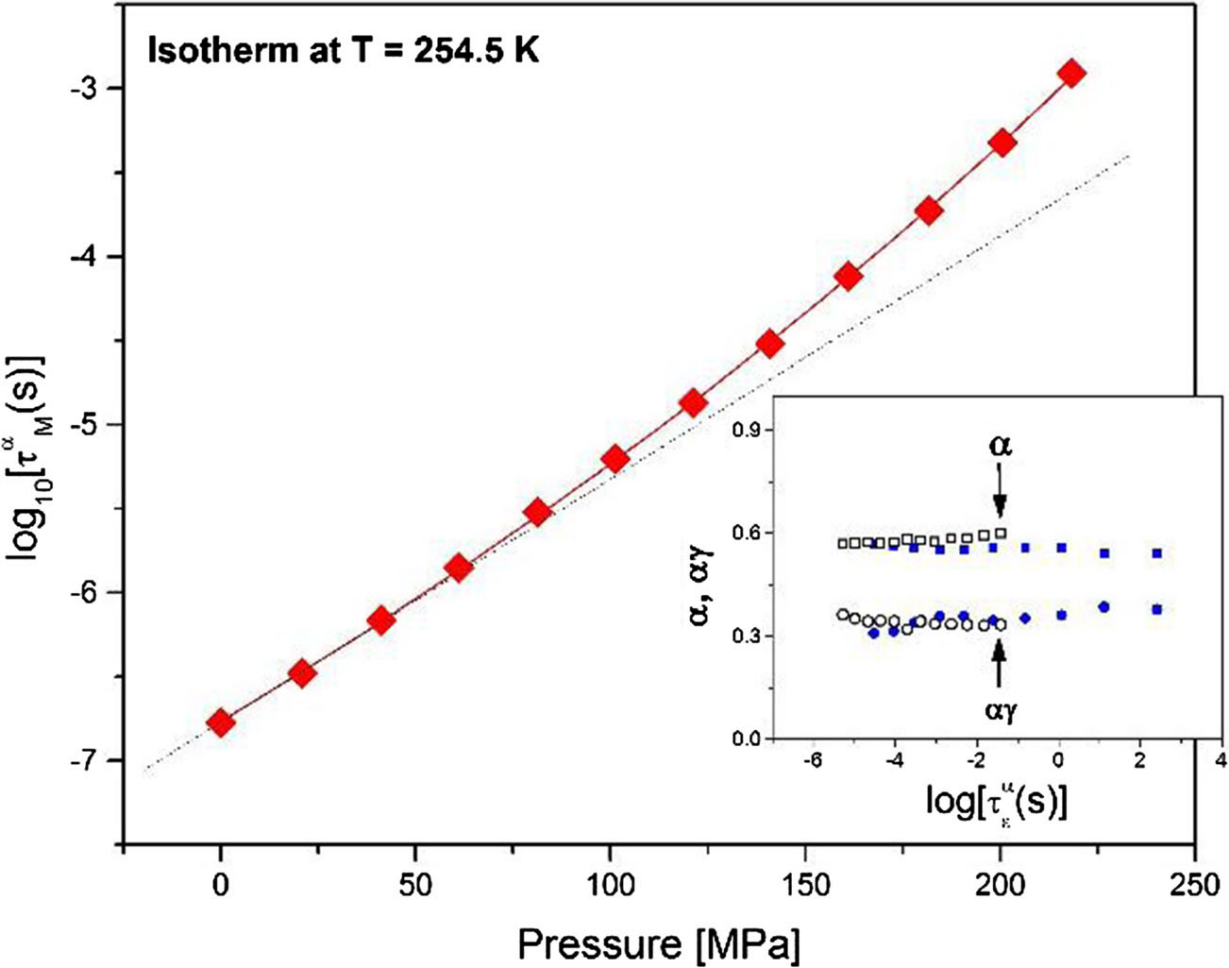
Pressure effect on the segmental relaxation time determined from the dielectric measurements. The *solid line* shows a fit of the relation (2) to the experimental data. *Inset*: parameters of the Havriliak-Negami function fitted to the frequency dependencies of the imaginary part of the dielectric permittivity measured both under isothermal and isobaric conditions

**Fig. 4:**
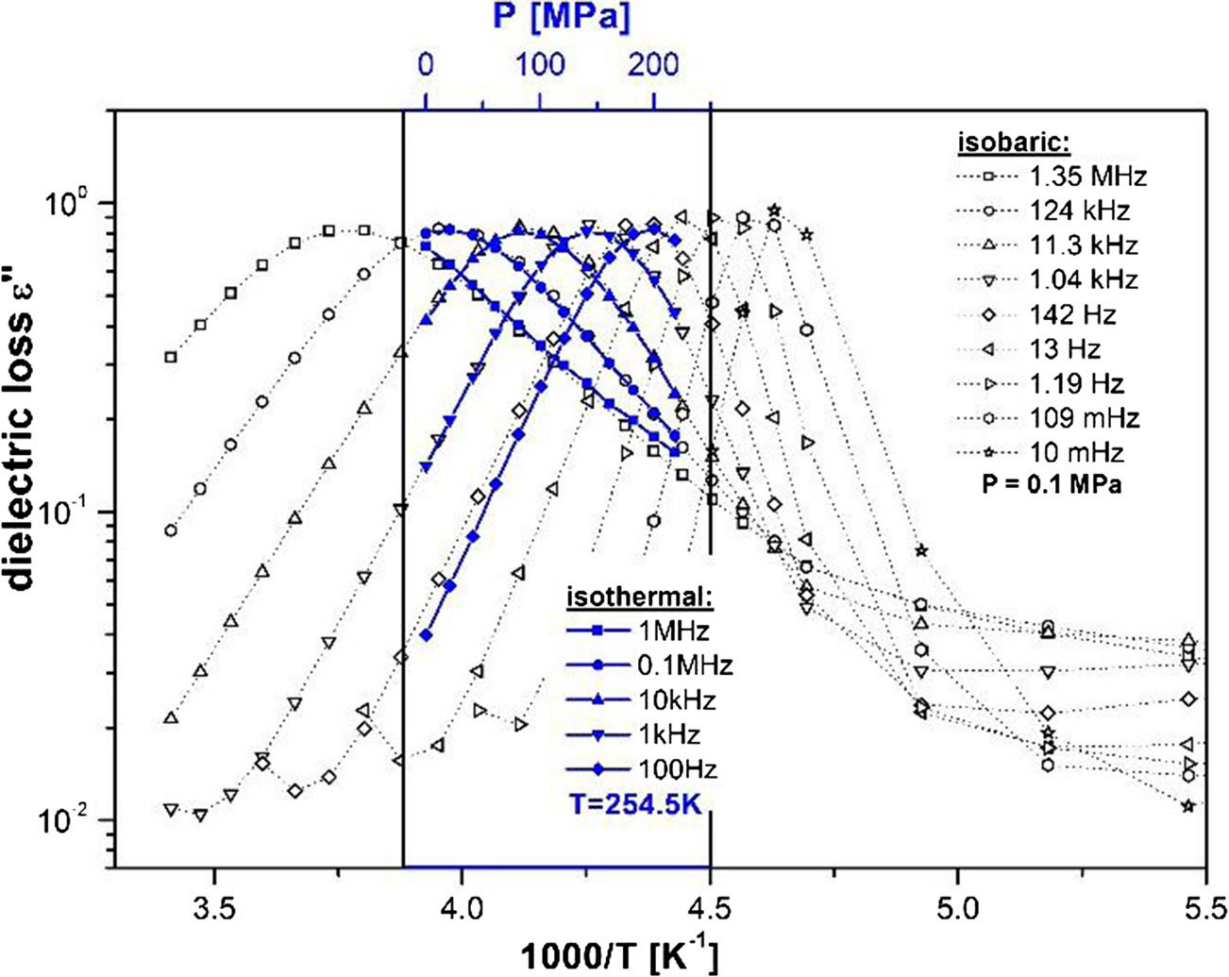
Examples of the imaginary part of the dielectric permittivity determined during the temperature or the pressure change with fixed frequencies

To obtain more information about the mechanism of segmental relaxation in the studied system, we introduced pressure as an additional variable. From Fig. 3, it can be easily seen that τ^α^_M_(P) exhibits a curvature analogous to the temperature dependencies of the segmental relaxation times. As a consequence, the simple volume activation model $\tau ={\tau}_0 \exp \left(\frac{ PV}{ RT}\right)$ cannot be applied to fit the experimental data [22, 23]. There are systematic deviations close to the glass transition, where segmental relaxation time is apparently more pressure-dependent than predicted by the above model. In our earlier studies, we demonstrated that pressure evolution of the α-relaxation process in low molecular glass-forming liquids could be well parameterized by means of the simple phenomenological expression [24]:

2 $$ \tau ={\tau}_0 \exp \left(\frac{D_PP}{P_0-P}\right) $$

where *τ*_0_ is the relaxation time measured under atmospheric pressure, *P*_0_ is the pressure of the ideal glass-transition, and *D*_p_ is a dimensionless parameter defined in analogy to the strength parameter *D*_T_ in temperature VFT law. Equation (2) allows us to effectively reproduce the experimentally determined pressure dependence of segmental relaxation times. The above result demonstrates the equivalence of the temperature and pressure paths for approaching a glassy state. It can be illustrated even more clearly when the straight comparison of the *1/T* and *p* dependencies of the *ε″* are collected in one figure (Fig. 4). Thus, we can conclude that that segmental mobility within the polymer chain of EHPB can be slowed down in the same fashion both by a change of the activation energy due to cooling and by a change of the intermolecular distances resulting from compression.

From the experimental data, we were able to roughly assess the value d*T*_g_/dP for EHPB as equal to 0.116 K/MPa which is significantly smaller than that obtained for 1,2-PBD (0.24 K/MPa) [9] and other vinyl polymers. On the other hand, from the ambient pressure data for *T*_g_ = *T*(*τ* = 1 s), we obtained the value of *m*_p_ = 85. In turn, these two quantities allowed us to estimate the value of the activation volume at *T*_g_ from the equation $\varDelta {V}^{act}=2.303 Rm\frac{d{T}_g}{ dP}$. For EHPB, we obtained the value of 0.19 dm^3^/mol. This value corresponds very well to the experimental value of 0.15 dm^3^/mol calculated directly from the analysis of τ(p) dependence using the following definition: $\varDelta V=2.303 RT{\left(\frac{d \log \tau }{ dP}\right)}_{T= const}$ for *T*_g_ = *T*(*τ* = 1 s). Smaller values of *dT*_g_/*dP* and Δ*V* ^*act*^ than the ones obtained for the 1,2-polybutadiene reflect the effect of hydrogen bonds on the dynamics of our polymer. However, by comparing with such well-known h-bonded systems as sorbitol (*dT*_g_/dP = 0.04 K/MPa) [25] or glycerol (*dT*_g_/dP = 0.035 K/MPa) [24], we can deduce that for EHPB h-bonds, the impact on dynamics, although not negligible, is much smaller than for small molecular systems.

**Fig. 5:**
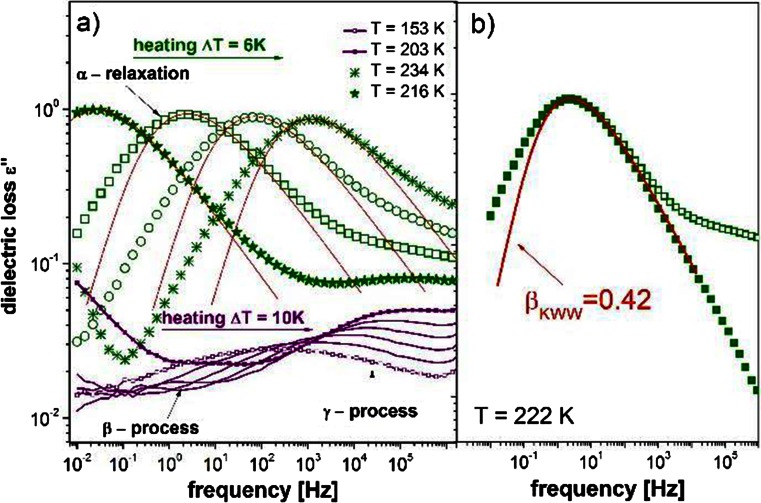
Dielectric spectra above *T*_g_ in the permittivity representation together with the KWW function with *β*_KWW_ = 0.42 describing the shape of all spectra. To show clearly the slope of the high-frequency side of the α-peak, spectrum recorded at 222 K with subtracted influence of the secondary relaxation is shown in panel b (*solid squares*). Below *T*_g_, two secondary relaxations (β and γ) are visible

As detailed above, below the glass transition temperature *T*_g_, in the region where the structure of the sample become frozen on the time-scale of the experiment, other relaxation phenomena, faster than α-process, can be detected in a dielectric spectrum. Usually as the temperature increases above *T*_g_, all the secondary processes tend to merge with the α-process at characteristic temperatures, forming only one process behaving as a continuation of either the α-process or the β-process from lower temperatures. However, for some samples, the existence of several active processes far above *T*_g_ is noted. Since secondary relaxations can originate in various molecular motions, they have been extensively studied in many glassy systems in recent years [26–29]. However, molecular mechanisms and the methods of studying them are still a matter of debate. The β-relaxation is the slowest and the most important of them. It is usually visible either as a well-separated peak or an excess wing at the high-frequency side of the α-peak [30]. Very often it can be detected solely below *T*_g_. It is called the JG-process, and unlike all the other secondary processes, it has been proven many times in the past to have an intermolecular origin both for small molecular and polymeric glasses [31, 32]. In our case, a small broad peak can be found in Fig. 1b. However, a closer look at the data obtained below *T*_g_ (Fig. 5) enables us to find another secondary process, slower than the one observed above the glass transition temperature. Thus, in the text below, we will use the term “γ-process” for the peak that is visible both above and below *T*_g_ and “β-process” for the one observed exclusively below *T*_g_.

Commonly, secondary relaxations are analyzed in permittivity representation. Although previously in this work we used modulus to compare the results obtained by dielectric and mechanical spectroscopies, there is no need to proceed with this representation, as the secondary processes are not visible in our mechanical spectra. Thus, in the latter part of this article, we will use permittivity representation. Not only will this enable possible comparisons with the data obtained for other materials but will also allow us to avoid the question of whether the analysis derived for permittivity representation is still valid in modulus formalism.

Figures 1, 2, and 5 allow one to observe the two most characteristic features of secondary relaxations. In Figs. 1 and 5, it can be seen that their strength decrease systematically with decreasing temperature. However, it becomes evident from Fig. 2 that the temperature dependence of characteristic secondary relaxation time in the glassy state follows the Arrhenius law:

3 $$ \tau ={\tau}_o \exp \left(\frac{E_A}{ RT}\right) $$

The values of the activation energy and the pre-exponential time constant obtained by fitting Eq. 3 to the experimental data are as follows: *E*_β_ = 58.15 ± 2.05 kJ/mol and logτ_0β_/s = −16.61 ± 0.57, *E*_γ_ = 36.8 ± 0.3 kJ/mol and logτ_0γ_/s = −15.77 ± 0.15, respectively. Some years ago, the Coupling Model predicting a correlation between the α and the JG-peak was derived. Within this model a simple equation [33]

4 $$ {\tau}_{\alpha}\left(T,P\right)={\left[{t}_c^{-n}{\tau}_0\left(T,P\right)\right]}^{\left(1/1-n\right)} $$

allows for calculation of the approximate relaxation time of the JG process, knowing the α-relaxation time and the α-peak’s shape parameter from the Kohlrausch-Williams-Watts function (β_KWW_ = 1 − *n*, where *n* is the coupling parameter) [34, 35]. This equation has been extensively tested in recent years both for neat systems and for mixtures since it has very important implications. On one hand, it allows one to determine whether the secondary process is of the JG type or is a conformational rotation within a single molecule [36–39]. On the other hand, since at the glassy state one is not able to track the α-process any longer, information about the relaxation of the structure can be gained by observing the behavior of the β-process (assuming the shape of the α-peak found in supercooled liquid regime does not change below *T*_g_). We also applied Eq. 4 to check whether any of the well-resolved secondary processes can be regarded as the Johari-Goldstein relaxation. The examples of the loss spectra with appropriate Kohlrausch-Williams-Watts (KWW) functions are shown in Fig. 5. To obtain the real slope of the primary relaxation at the high-frequency side of the peak, we subtract the influence of the secondary relaxation. The example of such a spectrum is shown in Fig. 5b. The relaxation times of the primitive process obtained from Eq. 4 are added to Fig. 2 as open triangles. Surprisingly, we found that the predicted relaxation times in the vicinity of the glass transition temperature agree very well with the relaxation times observed for the γ and not for the β-relaxation. In that case, one of two scenarios is possible. Either the JG relaxation does not need to be the slowest of the secondary processes or we are not able to appoint the true shape of the α-peak from the dielectric studies. Both reasons are likely. In this context, it is worth recalling that a similar discussion arose some years ago concerning descriptions of the secondary relaxations in decahydroisoquinoline. In this case, the γ not the β-relaxation was recognized as the JG process [40]. However, later on, basing on the DFT calculations, it was stated that probably either of these two relaxations was the JG-process [41]. Another example of organic glass for which at first glance the faster secondary relaxation was the JG process is the bis-5-hydroxypentylphthalate [42, 43]. Also in this case, superficial analysis failed, since it turned out that the shape of the α-relaxation was artificially broadened, predicting the false position of the JG process.

It was emphasized in the first section that the α-relaxation process in amorphous polymers does not satisfy a simple Debye model. The most commonly used empirical response function for the description of dielectric spectra data is the Havriliak-Negami function [44]:

5 $$ \varepsilon ={\varepsilon}_{\infty }+\frac{\varepsilon_s-{\varepsilon}_{\infty }}{{\left(1+{\left( i\omega {\tau}_{HN}\right)}^{\alpha}\right)}^{\gamma }} $$

where *α* and *γ* are fit parameters denoting the symmetric and asymmetric broadening of relaxation function.

Here, we applied Eq. (5) to extract shape parameters of the α-process, which was tested under both isobaric and isothermal conditions. To compare the effect of pressure and temperature changes on the shape of the response function, parameters *α* and *αγ* have been plotted on the inset panel to Fig. 3 as a function of log(τ_HN_). Evidently, the parameters remained invariant both with the change of the relaxation time and thermodynamic conditions. Such behavior is frequently described as the time-temperature-pressure superposition (TTPS) [45]. Similar behavior can be observed in other polymers as well as in low molecular glasses. Moreover, we observed that the peak of the segmental relaxation is very broad, which is a common situation for polymeric systems [46].

However, the most intriguing issue is that the peaks of the primary relaxation obtained for EHPB are clearly stretched, not only on the high-frequency but also on the low-frequency side. As can be seen in Fig. 3, the values obtained for the *α* and *γ* parameters are around 0.56 and 0.63, respectively (*α* · *γ* ≅ 0.35). The same values were obtained for pressure changes (open marks in the inset to Fig. 3). For polymer systems, a model to interpret the behavior of the shape parameters was proposed by A. Schonhals and E. Schlosser [47]. According to this model, the behavior of the *α* parameter (*ε″*(*ω*) *~ ω* ^*α*^ *for ω < ω*_*0*_) illustrates the influence of intermolecular interactions of segments of different chains, whereas the *α* · *γ* parameter (*ε″*(*ω*) *~ ω* ^-α*γ^ *for ω > ω*_*0*_) reflects intramolecular interactions between segments of a single chain. However, this low-frequency side stretching was not reported for all polymers: for example, for atactic polypropylene, polystyrene, poly(methylphenylsiloxane), and many others the left-hand side slope of the α-peak is equal to 1 [48–50]. All these examples feature with great randomness in dipole orientations along the chain. Thus, it was postulated that when the order in tacticity in the polymer system exists, then another process occurs. It is a bit slower than segmental relaxation but has very similar temperature characteristics. It is called a sub-Rouse mode [51]. It should not be identified with the normal mode present for polymers that have the dipole moment parallel to the chain backbone. Usually, this process has been observed by mechanical or photon correlation spectroscopy, and it is situated between the chain and segmental relaxations [52, 53]. However, Paluch et al. showed that the stretching of the low-frequency side of polymers is a sign that the sub-Rouse mode is also present in dielectric spectra [54]. From the examples the authors analyze in their paper, it becomes evident that for polymers for which sub-Rouse mode appears, the slope of the low-frequency side is around 0.4, as in our case. Thus, we can conclude that, in our material within sub-molecules not very much larger than segments, a correlation between dipole moments must exist. It induces the order in the tacticity in EHPB and causes the sub-Rouse mode to also be active in the dielectric spectrum. In accordance with previous observations of this mode, it must be a bit slower than segmental relaxation, but its temperature behavior is very similar to that of the α-process, since neither splitting nor merging of these two modes can be observed in dielectric spectra (the shape of the α-peak is invariant to temperature and pressure changes). Moreover, from our pressure studies, we can conclude that the pressure dependence of this mode is very similar to its temperature characteristic, since the spectra collected in pressure- and temperature-dependent experiments behave in a very similar fashion (see inset Fig. 3 and Fig. 4). Immediately, the question arises why we cannot observe this mode in the mechanical spectra. The reason lies in the fact that our polymer is not very long. Therefore, the peaks of chain and segmental modes lie very close to each other (see Fig. 1a). If we assume that the position of the sub-Rouse relaxation peak is the same in the dielectric and mechanical spectra, it becomes evident that it must be hidden under the high-frequency side of the Rouse peak and the low-frequency side of the segmental peak.

Another possible explanation of this unusual broadening of the left-hand side of the structural peak of the EHPB is the isotropization process that was observed previously for poly(n-alkylmethacrylates) by NMR techniques [55, 56]. It is caused by the loss of the conformational memory within the extended polymeric units. In the NMR spectra, it is visible as a process slower than the commonly observed segmental relaxation. The authors of the papers cited above introduced it as a “second aspect” of the structural process in polymethacrylates. In this context, it is worth mentioning that the time scale of this process corresponds quite well with our observations. It is slower but the difference between the relaxation times of this process and of the common αβ-relaxation is too small to allow for observation of two separate peaks. Moreover, polymethacrylates belong to the polymer family with stretched left-hand part of the dielectric loss spectra. However, to check for the above scenario in EHPB, NMR studies are needed since up to now it is the only method to successfully observe this intriguing process.

## Conclusion

We studied the dynamics of the epoxy/hydroxyl functionalized polybutadiene by means of dielectric and mechanical spectroscopies. We were able to observe several active modes both above and below glass transition temperature. In the mechanical spectra, we were able to recognize two processes which we described as chain and segmental relaxations. In the dielectric spectra, we distinguished five different relaxation processes. From comparison of the segmental modes obtained by dielectric and mechanical spectroscopy, we found that they have the same temperature dependence at higher temperatures, while they start to differ when approaching glass transition. We also observed the ionic conductivity process (visible as Debye peak in the lowest frequencies of the M″ spectra). Since the correlation between the α-relaxation and ionic relaxation is frequently discussed in literature, we also compared the temperature characteristic of these two processes. Again, we observed that they only have the same temperature dependence in the high-temperature region, while systematic deviations occur when the glass transition is approached. Such behavior was described also for non-polymeric systems, and it is caused by the fact that although the structure becomes frozen in the vicinity of the glass transition, small ions responsible for the ionic relaxation (or dc-conductivity) are still free to move. Moreover, in the glassy state, we found two secondary relaxations (β and γ). From the analysis by means of the coupling model, we found surprisingly that it is the γ relaxation that should be considered as a JG relaxation. There can be two explanations for this result. The first is that in h-bonded systems like EHPB, we cannot exclude the existence of some kind of clusters maintained by h-bonds with the relaxation dynamics positioned in between the primary and the JG relaxation. In this case, the JG relaxation would not be the slowest of the secondary relaxations. However, another explanation can also be given. One of the parameters in Eq. (4) is the shape of the segmental relaxation. However, in the case of EHPB, it is very difficult to determine this parameter very precisely, since it is probable that another peak is hidden under the left-hand side of the α-relaxation peak. Two possible explanations can be found in literature for such a process. One is the so-called sub-Rouse mode and the other is the isotropization process. Since it is not possible to say much about the shape and the temperature evolution of this new mode from dielectric studies, the shape of the primary relaxation can only be roughly estimated. Moreover, due to the fact that our polymer is not very long, the segmental and chain relaxations are not separated very much, and this prevents observation of the sub-Rouse relaxation in the mechanical spectra, where it is commonly seen when active. On the other hand, the isotropization process has only been observed up to now by NMR spectroscopy. Consequently, we are not able to unequivocally choose any of these scenarios. Notwithstanding, irrespective of the microscopic origin of this process, its possible existence in the dielectric spectra is very interesting and deserves further studies.
